# High-density surface electromyography signals during isometric contractions of elbow muscles of healthy humans

**DOI:** 10.1038/s41597-020-00717-6

**Published:** 2020-11-16

**Authors:** Mónica Rojas-Martínez, Leidy Yanet Serna, Mislav Jordanic, Hamid Reza Marateb, Roberto Merletti, Miguel Ángel Mañanas

**Affiliations:** 1 Department of Bioengineering, Faculty of Engineering, Universidad El Bosque, Bogotá, Colombia; 2 Biomedical Engineering Research Centre (CREB), Department of Automatic Control (ESAII), Universitat Politècnica de Catalunya (UPC), Barcelona, Spain; 3 Biomedical Research Networking Centre in Bioengineering, Biomaterials, and Nanomedicine (CIBER-BBN), Madrid, Spain; 4 Biomedical Engineering Department, Engineering Faculty, University of Isfahan, Hezar Jerib St., 81746-73441 Isfahan, Iran; 5 LISiN, Dept. of Electronics and Telecommunications, Politecnico di Torino, Turin, Italy

**Keywords:** Signal processing, Motor control, Biomedical engineering

## Abstract

This paper presents a dataset of high-density surface EMG signals (HD-sEMG) designed to study patterns of sEMG spatial distribution over upper limb muscles during voluntary isometric contractions. Twelve healthy subjects performed four different isometric tasks at different effort levels associated with movements of the forearm. Three 2-D electrode arrays were used for recording the myoelectric activity from five upper limb muscles: biceps brachii, triceps brachii, anconeus, brachioradialis, and pronator teres. Technical validation comprised a signals quality assessment from outlier detection algorithms based on supervised and non-supervised classification methods. About 6% of the total number of signals were identified as “bad” channels demonstrating the high quality of the recordings. In addition, spatial and intensity features of HD-sEMG maps for identification of effort type and level, have been formulated in the framework of this database, demonstrating better performance than the traditional time-domain features. The presented database can be used for pattern recognition and MUAP identification among other uses.

## Abstract (summary)

**Table:** 

Measurement(s)	muscle electrophysiology trait
Technology Type(s)	electromyography
Factor Type(s)	motor unit action potential • type of task • effort level
Sample Characteristic - Organism	Homo sapiens

Machine-accessible metadata file describing the reported data: 10.6084/m9.figshare.12808307

## Background & Summary

High-density surface electromyography (HD-sEMG) is a method for the recording of Motor Unit Action Potentials (MUAP) over a muscle, using 2D arrays of closely-spaced electrodes. Unlike traditional surface electromyography (sEMG), it accounts for both the spatial and temporal characteristics of the signal allowing a broader assessment of muscle electrophysiological activity. The recorded signal has three dimensions: two in the space and one in the time. This technique has gained attention during the last years for different applications such as signal decomposition (i.e., isolation and classification of individual MUAPs from the sEMG signal)^1^, the study of neuromuscular compartmentalization^2^, the analysis of changes in the spatial distribution of MUAPs with exercise or pain^3^, and pattern recognition for identification of movement intention^4^, among others. Despite the growing research interests, clinical applications and teaching remain limited.

This paper aims to describe and provide a database of HD-sEMG signals^5,6^ during voluntary isometric contractions of arm and forearm muscles of 12 healthy subjects. More than 336 signals per subject were acquired. This database aims to study patterns of sEMG spatial distribution over upper limb muscles during four tasks related to the forearm’s movements: pronation/supination and flexion/extension of the elbow. Tasks were performed at three effort levels (10%, 30%, and 50% of Maximal Voluntary Contraction). The database provides a foundation and a reference for, for example, studying patients with neuromuscular disorders or injuries, where the spatial intensity patterns can change with disease severity and level of recovery. It can also be used to test and validate signal processing or other techniques.

It represents an advance in state of the art thanks to the quality of the data and to the techniques used to verify their reliability. In previous studies, we have demonstrated that this database is useful for recognizing the isometric tasks of the upper limb. What is more, by using combinations of features based on spatial distribution (that is, in the spatial domain) and intensity of HD-sEMG^7,8^, it was possible to obtain higher performance in the classification than using traditional time-domain (TD) features or frequency-domain (FD) features (examples of these last can be found in^9^).

Additionally, this database has other potential applications. The main ones refer to teaching sEMG to clinical operators; using pattern recognition and machine learning techniques to identify movement intention from HD-sEMG, and testing algorithms developed for the decomposition of sEMG signals into the constituent MUAP trains to reveal control strategies adopted by the central nervous system. It can also be used to study regional inhomogeneities in the activation of motor units and local activation patterns in the upper arm and forearm muscles depending on the type of task and the effort level. Finally, other potential applications are the design and evaluation of methods for the automatic detection of innervation zones and the exploration of other spatial features to improve the identification of movement intention.

## Methods

### Participants

Twelve healthy male volunteers participated in the study (age, 28.3 ± 5.5 years; height: 177.8 ± 6.0 cm; body mass: 75.7 ± 8.7 kg). None had any history or symptoms of neuromuscular disorders, pain, or regular training of the upper limb. The information about protocols and possible risks related to the tests were given to every subject before signing an informed consent form. The tests were conducted in Italy following the Declaration of Helsinki and subsequent amendments concerning research in humans. They were approved by the Ethics Committee of UPC-BarcelonaTECH and the local Italian Health Delivery System. They were also supported and registered by the Spanish Innovation and Science Ministry (TEC2008-02754)

### Experimental protocol

**Fig. 1:**
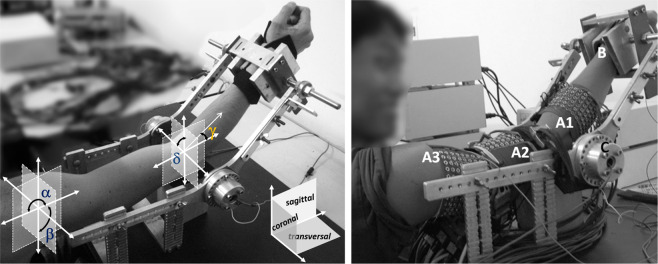
Subject position setup. Left. Position of the upper-limb during the experiment. The joint angles for the shoulder and the elbow are shown (reproduced from^8^ with permission from Elsevier). Right. Location of the electrode arrays 1–3 during the experiment (replicated from^7^).

The myoelectric activity was simultaneously recorded from five muscles of the dominant arm: biceps and triceps brachii, anconeus, brachioradialis, and pronator teres. During the test, subjects performed four different isometric tasks associated with supination/pronation and flexion/extension of the elbow. The arm and forearm were locked in a mechanical brace designed to measure isometric torques using two torque meters, one on the right and the other one on the left of the elbow joint and whose axes were aligned with the elbow rotational axis. Outputs of equal value and sign would indicate flexion or extension, while outputs of different signs would indicate supination or pronation (see Fig. 1).

Subjects were seated upright facing the mechanical brace with their back upright. Their dominant arm (dominance was indicated by the subject and was right in all cases) was parallel to the sagittal plane with the elbow flexed at 45° (*γ* = 45°), the shoulder abducted at 90°, and the forearm rotated 90°, so the thumb was facing upwards (Fig. 1). Each subject was previously trained to avoid activation of other muscular groups unrelated to the movements of the forearm. Besides, the wrist was fixed with an adjustable strap and a vice located at the distal end of the bars of the mechanical brace to avoid hand gripping.

Maximum Voluntary Contraction (MVC) was measured for approximately 3 s at the beginning of each test as the maximum of three consecutive trials for the four types of task, with two-minute rest between trials. Verbal encouragement was provided to produce a MVC. Subjects were asked to exert isometric contractions at 10%, 30% and 50% MVC for each task and the instructor supervised the correct execution of the task at all times. Each contraction lasted 10 seconds and was followed by two minutes of rest to avoid the effect of cumulative fatigue. The order of the contractions was randomized to prevent biasing effects.

### Data acquisition

Three 2-D electrode arrays were used for recording monopolar HD-sEMG signals. The electrode arrays were composed of equally spaced contacts separated by 10 mm in the x and y directions and made of silver-plated and gel-filled eyelets with 5 mm diameter. They met requirements for spatial sampling and for allowing interpolation^10,11^.

A moderately-elastic fabric was used as a substrate for eyelets. It allowed adapting the arrays to the shape of the muscle whereas preserving the inter-electrode distance within 0–10%. The substrate was hydrophobic and breathable to avoid possible electrical cross-bridges between channels caused by gel or sweat absorbed by the tissue.

**Fig. 2:**
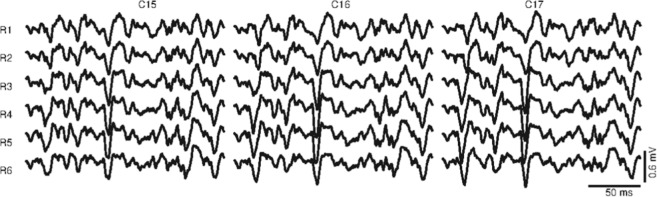
EMG monopolar signals of brachioradialis during a contraction at 30% MVC. The signals were recorded using Array 1. The figure displays the channels recorded over three columns (C15-C17) and six rows (R1-R6) in the original distribution of the array. It is possible to observe the propagation of Motor Unit Action Potentials along the muscle fiber direction, under the three columns of electrodes.

Array 1 (A1 in Fig. 1) was located on the forearm, with the first row 2 cm below the elbow crease, covering the muscles anconeus, pronator teres, and brachioradialis with at least four columns of electrodes each. The edges of these muscles were previously drawn on the surface of the skin following the guidelines proposed in^12^. Array 1 had six rows, and the number of columns read by the acquisition system was commuted between 16 and 19 columns of electrodes, depending on the forearm circumference. Arrays 2 and 3 were located at the distal and proximal regions of the upper arm (see Fig. 1), covering the muscles biceps brachii and the upper part of the triceps brachii, respectively. These arrays were placed to cover the sensor location recommended by SENIAM^13^ (www.seniam.org) (for details, please refer to the next section). Both arrays (A2 and A3) had eight rows and 15 columns of electrodes. The skin was shaved and cleaned with abrasive paste^14^. Contact was improved by inserting 20 μl of conductive gel in each eyelet with a pipette to reduce electrode-skin impedance. Figure 2 displays an example of the signals recorded in three columns and six rows of Array 1 (forearm). It is possible to observe the EMG signals of the brachioradialis during flexion at 30% MVC over a window of 200 ms.

For comparing muscle activation areas among subjects, the lengths and circumferences of the upper forearm and arm were measured as follows. The length of the forearm was measured from the medial epicondyle to the epiphysis of the radius. The length of the ventral face of the upper-arm was measured from the acromion to the fossa cubit. The length of the dorsal face was measured from the posterior crista of the acromion to the olecranon. Circumferences of the arm segments were measured while contracting different muscles: the proximal forearm circumference was measured 2 cm below the elbow crease, and the distal and proximal upper arm circumferences were measured over the muscle belly of biceps and triceps respectively.

Three amplifiers (OT Bioelettronica EMG-USB-128 channels, with a sampling frequency of 2048 Hz, a 3 dB bandwidth 10–750 Hz, programmable gains of 100, 200, 500, 1000, 2000, 5000 and 10000, CMRR >90 dB, and input impedance >300 MΩ at 50 Hz) were used to simultaneously record monopolar sEMG signals with synchronized sampling provided by an external clock. Common mode interference was reduced by using a “driven right leg” (DRL) circuit^15^ with reference and feedback electrodes placed at the clavicle, wrist, and shoulder of the subject’s dominant side. A virtual ground^16^ was used to enhance the quality of the monopolar signals. Power line interference (50 Hz) was strongly limited but not fully cancelled; additional data processing may be necessary to reduce it further.

**Fig. 3:**
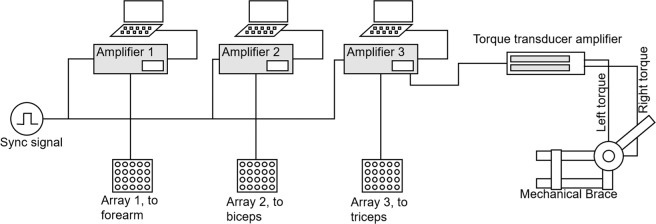
The instrumentation set up for the experimental protocol. In the case of elbow flexion-extension, the two torque transducers on the two sides of the arm provided equal signals in the case of extension or flexion of the elbow. In the case of pronation-supination, they provided opposite signals. This information was used to help the subject produce the correct effort. The two force signals were displayed using two bars of LEDs that provided visual feedback to the subject.

Figure 3 shows the instrumentation setup. The amplifiers 1 and 2 recorded signals from the forearm muscles and biceps brachii muscle, respectively. The amplifier 3 recorded signals from the triceps brachii muscle and the two torque signals sensed by two torque transducers (OT Bioelettronica, range 150 Nm, supply voltage = ±5 V, full range = 25 mV) located at the joints of the mechanical brace and aligned with the elbow rotational axis (Fig. 1). The torque signals were amplified, recorded, and displayed in real-time to provide the subject with visual feedback of the produced force.

### Reference system

A reference coordinate system was defined for each muscle to standardize the recording electrodes location among subjects. The abscissa and ordinate axes (*x,y*) were set parallel to the medial-lateral and the proximal-distal directions, respectively, and normalized by the circumference and length of the limb segment related to each array as explained in^7^, see Table 1. The origin of the coordinate axes coincided with the sensor location recommended by the SENIAM project and was defined as:

- Array 1: a point located at the intersection between the line that connects the origin and insertion of each forearm muscle (anconeus, pronator teres and brachioradialis) and the forearm arc located 2 cm below the elbow crease.
- Array 2: a point located at 3/4 the distance from the origin of the biceps brachii to its insertion over the line that connects these two points.
- Array 3: a point located at 1/2 the distance over the line that connects the origin and insertion of the lateral head of the triceps brachii.

**Table 1:** Anthropomorphic data measured to standardize the upper-limb size among subjects.

Array	*x*-coordinates Circumference	*y*-coordinates Distance
1	Proximal forearm	Medial Epicondyle - Process of Radius
2	Distal upper-arm	Acromion - Fossa Cubiti
3	Proximal upper-arm	Acromion - Olecranon

For each array, circumferences (for the *x*-coordinates) and longitudinal distances (for the *y*-coordinates) were measured at different upper-limb segments. For arrays 2 and 3, distances were measured considering the reference points proposed by the SENIAM project^13^. See text for the definition of the origins.

## Data Records

Data records presented in this section and accompanying detailed description file (README) are available online from *figshare*^5,6^. The records contain the raw signals without any further processing. Data are stored in individual folders for each of the twelve subjects (s1-s12). Every subject’s folder contains four subfolders, one with the torque signals and the other three with files of the signals registered by each array: *forearm* subfolder for the array 1, *biceps* subfolder for the array 2 and *triceps* subfolder for the array 3. Files, in ascii format, are named according to the type of task and effort level.

Additionally, the database includes the following files:

- **ReferencePoints.txt**: provides information about the final location of each array on the upper arm or forearm. The data is presented in table form. Each row corresponds to one subject in the database, and each column shows the distance in cm from the reference (origin of the coordinate system of the muscles) to the first electrode locating in the upper-left corner of the array. This information is consigned for the x and y axis.
- **nchannels.txt**: provides the number of channels registered by subject and array.
- **forearm.txt**: contains the channels (first and last) covering each of the muscles of interest in the forearm for every subject.
- **SubjectsDescription.txt:** gives details of the population’s age, height, weight, and dimensions of the limbs. Length and circumference were measured, as depicted in Table 1.

## Technical Validation

The methods described herein were applied in previous studies to the presented database. However, the reader must bear in mind that the shared dataset comprises the original monopolar raw signals so they can be used for testing new processing methods. The raw signals were collected with the DRL technique and analog filtered between 10–750 Hz. In the subsequent sections, the different signal processing methods used for the detection of atypical signals (artefacts), calculation of activity maps, and identification of volitional movement intention, are briefly described. As well, the original references where the methods were described in depth are provided^4,7,8,17^.

### HD-sEMG signals quality: outlier detection

In HD-sEMG recording, examining the electrode-skin contact quality before signal recording is not always a practical task. Considering that the protocol consisted of the simultaneous recording of many signals (approximately 340 per subject, task, and effort level), it was expected to observe bad quality channels or outliers mainly caused by poor electrode-skin contacts. Besides, some cable and skin movement artefacts are usual when recording sEMG signals, even in isometric conditions.

For detecting bad quality signals, two different methods were proposed: the first was supervised and based on an expert system as described in^7^ and the second consisting of a non-supervised method employing local distance-based outlier factor^17^. For the detection of outliers, each channel was characterized by a set of features inspired by experts’ criteria to classify “good” and “bad” channels:

1. The relative power of low-frequency components $({P}_{\frac{l}{t}})$, from 0 to 12 Hz. This feature is associated with movement artefacts that mostly cause large and slow transients in the signals.
2. The relative residual power of power-line interference $({P}_{\frac{line}{t}})$, corresponding to 50 Hz and its first four harmonics. Power line interference is caused by poor contact between the electrodes and the skin.
3. The signal power estimated from the root mean square (RMS) of each signal calculated over epochs of 500 ms. This was done to identify channels with much higher or lower power than that of neighboring channels, and finally
4. The similitude between adjacent (horizontal, vertical and diagonal) channels measured from the average cross-correlation coefficient.

Channels with values exceeding a threshold over any of these features were considered outliers.

Given that the arrays may lie over regions with high and low activation simultaneously (for example, if the array is covering different muscles), the outlier detection methods refer these features to the neighbor channels rather than considering the bulk of data.

**Fig. 4:**
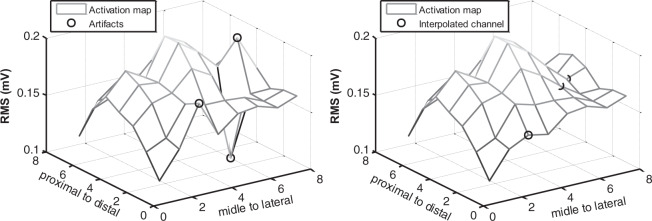
Substitution of “bad channels”. Figure reproduced, with permission, from^5^. The outlier channels were replaced with channels obtained by the interpolation of the neighbors.

Because the first method for detecting outliers was supervised, its performance was better than the second one, so its results are described hereon to estimate the quantity of expected low-quality signals in the database. The proportion of outliers varied between 0 and 13% for each sample in the training set (one sample corresponding to the signals recorded by one array) according to the opinion of three different experts^7^. The expert system reached a precision of about 95% and a sensitivity of 93%. Overall, approximately 6% of the total number of recorded signals (that is, 3045 of 50760 sEMG signals recorded in total for 12 subjects, four tasks, and three contraction levels) were identified as outliers (6.3% ± 2.9% per subject). Taking into account that the outlier detection algorithm in^7^ showed an outstanding performance in the validation set, it is possible to say that the quality of the signals in the database is excellent. An example of how the bad quality signals can affect the analysis of the spatial distribution of the MUAPs is presented in Fig. 4 (left). However, bad quality channels can be corrected by the identification of artefacts, Fig. 4 (right).

### Analysis of the spatial distribution of HD-sEMG intensity

HD-sEMG provides insights into the spatial distribution of myoelectric signal intensity over the muscle. Spatial distribution of muscle activation can reflect important information on the properties of the muscle, such as fatigue^18,19^, exerted force^20,21^, and joint position^22^.

This database was used to evaluate patterns in spatial distribution during upper limb tasks. It was observed that specific spatial patterns are typical for all subjects and each level of effort. That is, there are unique and repeatable activation patterns that are specific for each task and each effort level, and these activation patterns are common for all subjects. These studies and conclusions were published in^7,8^ and were the basis of a follow-up study where the repeatability of the spatial and intensity patterns was evaluated in patients with incomplete spinal cord injury^23,24^.

Spatial patterns were characterized using activation maps (AM). A representation of the HD-sEMG monopolar signals recorded in two dimensions as images, where pixel locations correspond to positions of electrodes in the array, and pixel intensities correspond to intensities (RMS estimated over non-overlapping epochs of 500 ms) of monopolar signals in corresponding channels. They provide a global view of muscle activity in a broad region by quantifying the intensity of the sEMG signals and its spatial distribution over the muscle. Each AM is calculated as:

1 $$A{M}_{i,j}=\sqrt{\frac{1}{N}\mathop{\sum }\limits_{n=1}^{N}sEM{G}_{i,j}^{2}\left(n\right)}$$

where N corresponds to the number of samples in each epoch (1024 samples) and *sEMG*_*i,j*_ denotes the sEMG signal recorded by the electrode located at row *i* and column *j* in the recording array. For the calculation of the activation maps the sEMG signals were previously band-pass filtered between 12 and 350 Hz with a 4th order digital Butterworth filter in forward and backward direction according to SENIAM recommendations for the processing of surface EMG signals^13^.

**Fig. 5:**
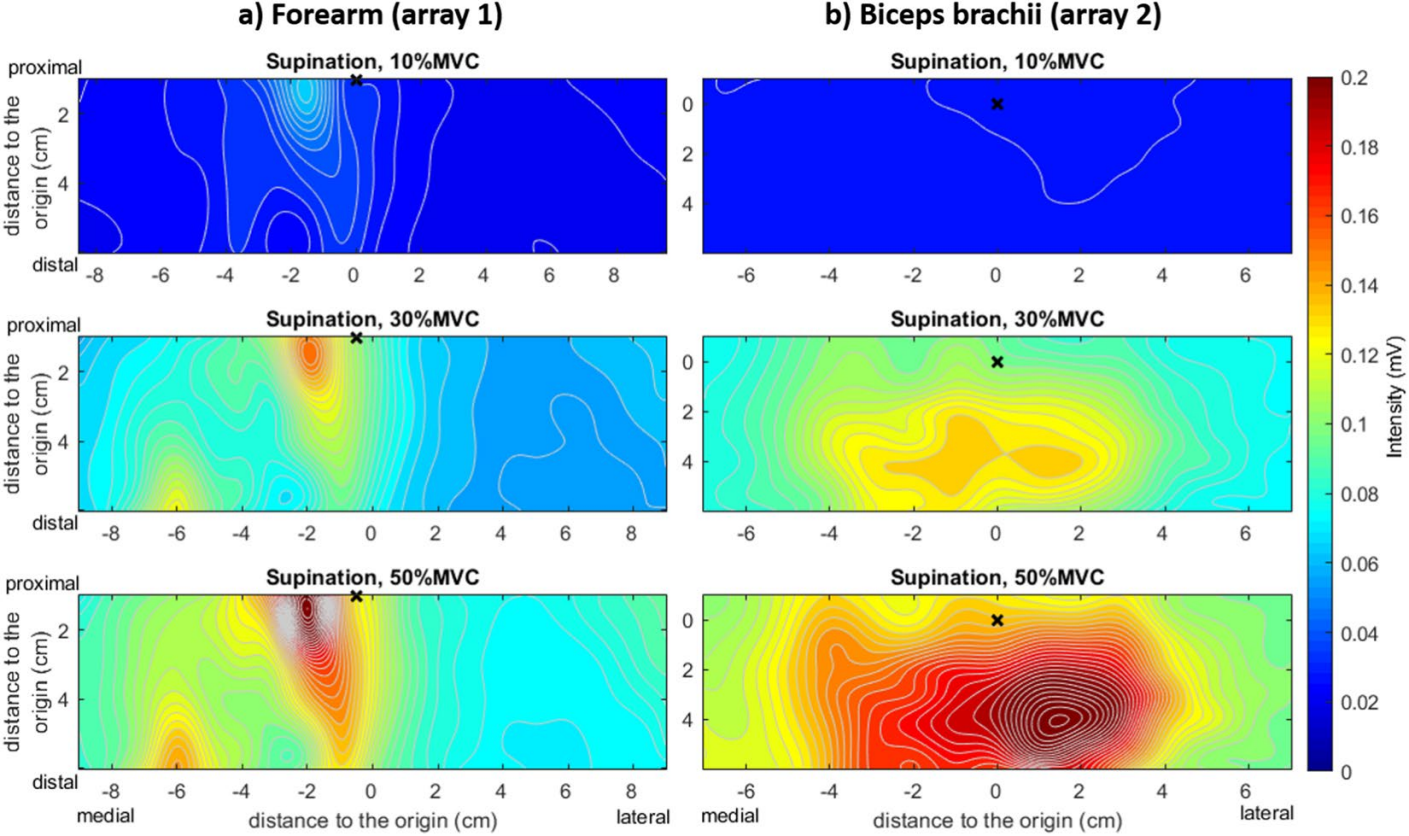
Monopolar activation maps for subject 8 in the database during supination isometric efforts. The maps were averaged for segments of 3 s using six epochs of 500 ms. The anatomical references, that is, the origin of the reference system (0, 0) for each map as described in the “Reference System” section is displayed with a cross (×) (**a**) Forearm. In the case of the forearm, the reference for the anconeus is shown. The brachioradialis is located at the left of the reference, and the Pronator Teres is located at the right. (**b**) Biceps Brachii. It is possible to observe that the intensity increases with the effort level (warmer colors represent higher intensity) and that the spatial distribution also changes (see contour lines).

Figure 5 shows an example of a forearm and biceps AM for one of the subjects in the database. In this case, the maps correspond to isometric supination efforts of the forearm at 10%, 30% and 50% MVC. It is possible to see the variation of the intensity and distribution under the electrode grid as a function of the effort level. Every AM was obtained by averaging six consecutive maps from six non-overlapping time segments of 500 ms (N = 1024 samples)^7,8^. The 3 s interval was selected as the period of the greatest force stability in the 10 s recording.

To define the regions of activity associated with each muscle, segmented AM were calculated for all cases. Segmentation of the active areas (i.e., areas with the highest intensity) was performed over each AM by applying an h-dome transformation^25^. This transformation is especially important for the array A1 were signals from three different muscles were simultaneously recorded.

Segmentation discards the map areas of low intensity (low RMS) and divides the forearm map into three different regions, each of them corresponding to one forearm muscle. Segmentation allows the analysis of the areas associated with each muscle (triceps brachii, biceps brachii, brachioradialis, anconeus, and pronator teres).

**Fig. 6:**
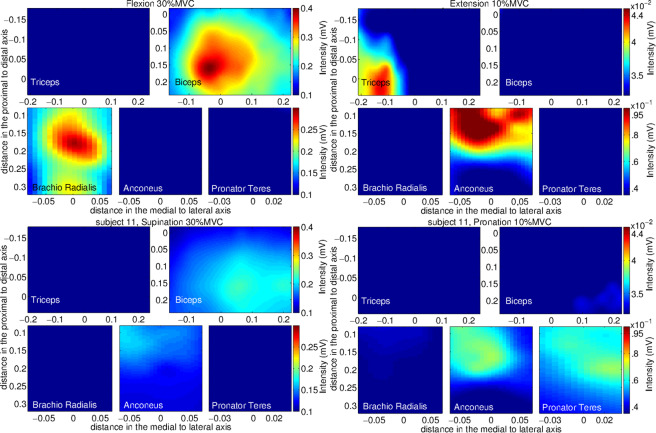
Segmented maps obtained from one of the subjects for the five muscles under study: triceps brachii, biceps brachii, brachioradialis, anconeus, and pronator teres. The maps were averaged for segments of 3 s using six epochs of 500 ms. The four tasks carried out during the test are shown: flexion at 30% MVC (top-left), extension at 10% MVC (top-right), supination at 30% MVC (bottom-left) and pronation at 10% MVC (bottom-right). Distances are presented as fractions of the arm circumference (x-axis) or segment length (y-axis). Differences in the average intensity as well as in the spatial distribution can be observed in all maps. The maps are represented in the reference system defined for each muscle (see text) RMS values are calculated over a 500 ms epoch and interpolated by a factor of 100. Reproduced from^8^ with permission from Elsevier.

Figure 6. shows an example of monopolar segmented AM obtained from one of the subjects for the five muscles and the four motor tasks at two different effort levels. It can be seen that the shape of the segmented area depends on the intensity of the peaks. The segmentation facilitates the identification of areas associated with the contraction of each muscle by selecting the regions of higher energy. This procedure diminishes confounding factors resulting from the synergistic contraction of adjacent muscles.

### Identification of task and effort level using HD-sEMG signals

This section presents an example of the application of the stored signals.

One of the most critical applications of the sEMG recording is the control of prosthetic, assistive, or external devices. Following previous findings on changes in the spatial distribution of HD-sEMG associated with tasks and effort levels, a method for automatic identification of movement intention was developed and tested.

To prove that the data allow the recognition of forearm efforts in isometric conditions, several features proposed by the authors have been evaluated in the a) identification of tasks and b) identification of tasks and effort levels.

**a) Identification of the tasks** corresponds to the identification of movements at the elbow joint (flexion/extension and supination/pronation). Corresponding identification classes are flexion, extension, supination, and pronation. All recordings were used in the task identification, regardless of the effort level at which every recording was performed.

Identification of motor tasks was performed using the linear discriminant classifier (LDC)^4^ and several types of intensity and spatial features (defined in the following paragraph). The classification procedure was evaluated using the repeated holdout method (N=20)^26^, where observations were randomly assigned to either the training set or the test set (70% and 30% respectively). Approximately, an equal proportion of samples of each class (flexion, extension, supination, pronation) was assigned to the training set and the test set; thus, yielding balanced training and test sets^27^. Results were reported in terms of the area under the receiver operating characteristic (ROC) curve (AUC), the Accuracy (ACC) and the F1 score (F1), defined for each class as:

2 $$ACC=\frac{TP+TN}{TP+TN+FP+FN}$$

3 $$F1=\frac{2\ast TP}{2\ast TP+FP+FN}$$

where *TP* represents the number of true positives (samples correctly classified to a specific class), *TN* the number of true negatives (samples correctly classified as negatives to a specific class), *FN* the number of false negatives (samples belonging to the observed class, but erroneously associated to another), and *FP* the number of false positives (samples belonging to another class but incorrectly associated to the observed class)^28^.

Results of task identification were compared using four feature sets extracted from epochs of 150 ms. Features were calculated for each muscle separately and then concatenated to form a feature set. The length of the epoch (150 ms) was selected as the shortest time segment before a decrease of the identification rate. The feature sets are described in the following paragraphs.

1. **Intensity features (I)**
  This feature set was composed of the intensity (I) of a segmented region of the activation map *AM* for each muscle containing M pixels (for details on how the segmentation was performed, please refer to^8^ calculated as:
  4 $$I={{\rm{\log }}}_{{\rm{10}}}\left(\frac{1}{M}\sum _{m}A{M}_{m}\right)$$
  This equation resulted in a single value calculated from a single activation map. The feature set formed an array of five intensities corresponding to the five muscles obtained by concatenation of intensities calculated from AM of individual muscles.
2. **Combination of intensity and mean shift features (IMS)**
  This feature set is a combination of the intensity feature (I) and spatial information extracted from AM using the mean shift algorithm^29^. This algorithm is a non-parametric approach to identify local maxima of the probability density function (pdf) of the amplitude of the pixels. After random initialization in the feature space, the algorithm iteratively searches for the peaks in the density function by taking steps in the direction of local gradient of the density function. This gradient is estimated by taking into account the samples located within the prespecified bandwidth of the current location. A detailed description of the algorithm to calculate this spatial feature can be found in^4^.
  To obtain the combination of features, the five intensities, relative to the five muscles and calculated as described in the previous paragraph, were concatenated with the mean shift features. The mean shift features of each array were also concatenated, and the dimensionality of this combination was then reduced using principal component analysis (PCA). Only the transformed components describing more than 90% of the cumulative variance were kept.
3. **Combination of intensity and center of gravity (ICG)**
  This feature set is a combination of intensity feature (I) and centers of gravity of AM, which provide an indication of the location of the region of activity (initially proposed in^8^).
4. **Classical time-domain feature set (TD)**
  This feature set is a combination of the time-domain features that are most often used in the literature^30^. This combination consists of RMS value, mean absolute value, number of zero crossings, waveform length, and number of slope sign changes calculated for all channels and all muscles in the 150 ms epoch. The dimensionality of the obtained feature vector was reduced using PCA transform. Only the components explaining at least 90% of the variance were kept.

**Fig. 7:**
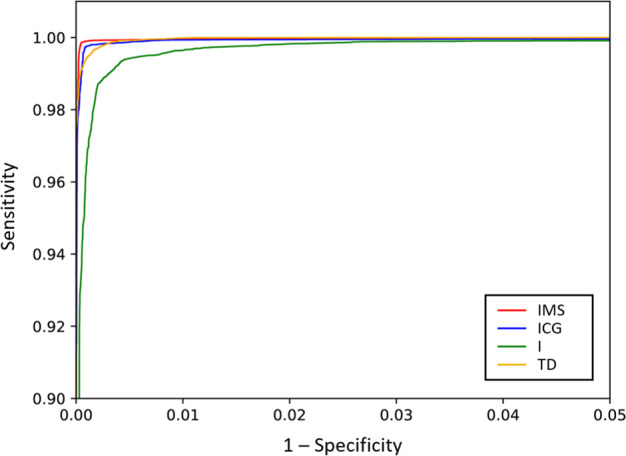
ROC curves for the identification of task (flexion, extension, pronation or supination) using intensity and mean shift (IMS), intensity and center of gravity (ICG), the intensity only (I), and time-domain (TD) features. The identification was evaluated using the repeated holdout method (N = 20) for each of the 10 subjects, and so, the plots represent 200 executions in total. Note the limited range of both x and y axes.

Figure 7 shows the ROC curves for the task identification using these four feature sets as obtained during the validation with the repeated hold-out method^4^. The area under the curve (AUC) for each set was as following: AUC_IMS_ = 99.8 ± 0.3%, AUC_ICG_ = 99.7 ± 0.49%, AUC_I_ = 99 ± 1% and AUC_TD_ = 99.6 ± 0.7%. The best classification was achieved for the features IMS with an ACC_IMS_ = 99.9 ± 0.14% and F1_IMS_ = 99.8 ± 0.3%.

**b) Identification of tasks and effort levels** corresponds to the simultaneous identification of task (i.e., flexion, extension, supination, or pronation) and level of effort (i.e., 10% MVC, 30% MVC, or 50% MVC). Consequently, 12 different classes (four per task by three per effort level) were considered (Table 2 lists all classes).

**Table 2:** List of the classes. In the identification of tasks, there are four classes, whereas in the identification of tasks and effort levels there are twelve different classes.

Identification of tasks	Simultaneous identification of tasks and effort levels		
	10% MVC	30% MVC	50% MVC
Flexion	Flexion 10% MVC	Flexion 30% MVC	Flexion 50% MVC
Extension	Extension 10% MVC	Extension 30% MVC	Extension 50% MVC
Supination	Supination 10% MVC	Supination 30% MVC	Supination 50% MVC
Pronation	Pronation 10% MVC	Pronation 30% MVC	Pronation 50% MVC

**Fig. 8:**
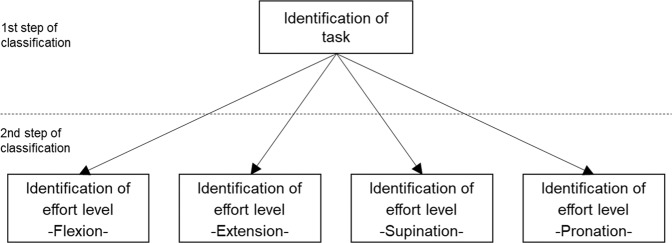
Scheme of the 2-step process for the identification of task and effort level. In the first step, the task was identified, and in the second step, the effort level was identified using one of four classifiers. The selection of a classifier in the second step was based on the result of the classification of the first step. For instance, if the task of a sample is identified as *flexion* in the first step, the effort level is identified using the classifier dedicated to the identification of effort levels for flexion task (the bottom left block in the figure), and if the sample is identified as *pronation*, the effort level is identified using the classifier for identification of effort levels for pronation task (the bottom right block in the figure).

The simultaneous identification of task and effort level was carried out by using a 2-step process based on the LDC. **In the first step**, only the task was identified regardless of the effort level, as described in the previous section. In this case, recordings of different effort levels were pooled in a single class (i.e., flexion, extension, supination, and pronation). In the second step, the effort level was classified. There were four different classifiers in the second step, each classifying effort level (10% MVC, 30% MVC, or 50% MVC) for a specific task. The identification of a sample in the first step (identification of task) determines the selection of a classifier for the second step (identification of effort level). The classification scheme is displayed in Fig. 8.

**In the second step**, only the features associated with agonist-antagonist muscle pairs involved in the task identified in the first step were used to classify the effort level (i.e., 10% MVC, 30% MVC and 50% MVC). That is, biceps brachii and triceps brachii for both *flexion* and *extension*; biceps brachii, brachioradialis and anconeus for *supination*; and pronator teres and anconeus for *pronation*. These muscles were selected following the procedure described in^8^ and^7^ on the same database.

**Fig. 9:**
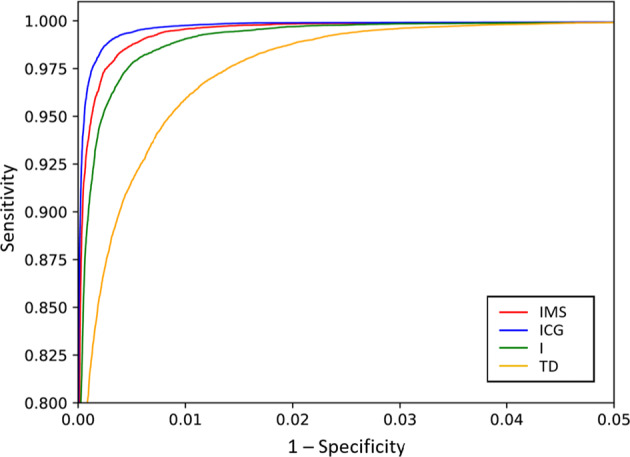
ROC curves for the Identification of short-term identification of task (flexion, extension, supination and pronation) and effort level (10%, 30% and 50% MVC) using intensity and mean shift (IMS), intensity and center of gravity (ICG), the intensity only (I), and time-domain (TD) features. The identification was evaluated using the repeated holdout method (N = 20) for each of the 10 subjects, and so, the plots represent 200 executions in total. Note the limited range of both x and y axes.

The ROC curves for the identification of tasks and effort levels are shown in Fig. 9. Results show that the features based on spatial information (IMS and ICG) outperform the other feature sets. In this case, the AUC for each feature set are as following: AUC_IMS_ = 97,5 ± 4%, AUC_ICG_ = 98,2 ± 3.6%, AUC_I_ = 96,4 ± 6,8% and AUC_TD_ = 94,6 ± 4.8%. In the case of the features based on spatial information, the Accuracy and the F1- score were as following: ACC_IMS_ = 99.6 ± 0.4%, ACC_ICG_ = 99.7 ± 0.3% and F1_IMS_ = 97.3 ± 2.4%, F1_ICG_ = 98.1 ± 2.1%.

The presented results, previously reported in^4^, demonstrate that the data can be used for the identification of isometric tasks associated with upper-limb and the levels of effort, even at very low contraction such as 10% MVC. In particular, combinations of features based on spatial distribution and intensity of HD-sEMG (IMS and ICG) have shown to yield a significantly higher identification rate (p < 0.05; Wilcoxon signed-rank test) than the traditional ones (i.e., I and TD) in all comparisons presented.

## Limitations

We are aware that isometric contractions are not representative of muscle activation for some practical applications. However, isometric conditions like the ones assessed with the presented database, are particularly suitable to test signal processing algorithms or to define new feature sets because possible sources of errors such as the relative movement between the muscle and the recording electrodes are small. New features in the spatial domain^4,8^ were presented and validated with this database and were successfully applied in a more practical application with dynamic tasks^31^. Moreover, maximal voluntary contraction was recorded as a reference for further processing and comparison of data between different recordings and/or between subjects. Although this is a recommended method in the population of healthy volunteers, it may not be suitable for subjects with an injury recovery process since they may not be able to exhibit a maximal contraction^32^. Furthermore, although there is evidence that the method used to measure maximum voluntary contraction is valid (i.e. with the arm fixed on a table), activation of the trunk or shoulder muscles may affect the sEMG signal of the arm muscles^33^. The signals were recorded in monopolar montage considering that in this way it was possible to detect the entire information contained in the signal, and so, the signals are expected to be contaminated by crosstalk^11^. However, since no spatial filters were applied during the recording, it is possible to reduce crosstalk offline by applying these kinds of filters in any direction. Another limitation of this study is the limited number of subjects. Care must be taken when using the database for example for classification.

## Usage Notes

The database can be used for different purposes. The main one is the application of pattern recognition and machine learning procedures to improve the identification of movement intention from sEMG maps, by mostly exploring spatial features either in amplitude or frequency domains. Other applications can be:

- To test algorithms intended for the decomposition of sEMG signals into the constituent MUAP trains for examining central nervous system strategies. For example, for different contraction levels and tasks. One example can be the use of the CKC algorithm intended for HD-sEMG^34^.
- To study regional inhomogeneities in the activation of motor units in the upper arm and forearm muscles and to consider how these changes with different levels of effort and myoelectric fatigue^21,35^.
- To study regional activation patterns during selective contractions for biofeedback purposes, especially in the case of forearm muscles (see, for example,^36,37^).
- To test and design methods for the automatic detection of innervation zones and the propagation of MUAPs^38,39^.
- To test the robustness of classification in case of smaller electrode grids and the sensitivity to electrode shift.
- To study myoelectric manifestations of muscle fatigue.
- To test outliers’ detection and cleaning methods in EMG^40,41^

## Data Availability

The custom code used for reading the signals of the database was created in MATLAB R2017b and is freely available at *figshare*^42^ or at the GitHub repository https://github.com/lyanet-upc/hd-emg-app.git. We provide: A readme file (**readmeapp.txt**) with instructions about how to run the code in a 2017b or higher Matlab version. A zip file (**hd_emg_app.rar**) containing: –the code main function (**app_hd_emg.m**). This function deploys an interactive Matlab app from which users can load, and friendly visualize data of a specific subject. Here, parameters like the type of task, effort level and signal window size can be set easily. Plots of AM and sEMG of all or a specific channel are provided and can be modified by selecting different times. –a function folder with auxiliary functions (**read_hd_emg_signals.m, get_color_scale.m, plot_hd_emg_maps.m**) needed to run the main function. A Matlab script (**db_reader.m**) with a simple example about how to read and plot data of a specific subject, tasks, effort level and muscle using Matlab code.
